# The Treatment of "Dull" and "Defective" Children

**Published:** 1913-11-08

**Authors:** 


### THE SCHOOL DOCTOR'S DIFFICULTIES.

### I.—The Treatment of "Dull" and "Defective" Children.

There are few more thankless tasks than the selection of children for special schools for mentally defectives, yet there are few also that exact more time, care, patience, and tact from the school doctor. He is as it were between two fires. In present circumstances no teacher cares to retain in his or her class a child who is dull or backward. The standard exacted by the Board of Education is so high—needlessly high, we think—and that set by certain education authorities (for instance, the London County Council in regard to scholarships) is equally high—that the teacher must slave unduly hard and devote a disproportionate amount of individual attention to a child who is not "average" so far as intelligence is concerned. Consequently, teachers are only too prone to ask the doctor to pass as "mentally defective" children who are not, strictly speaking, mentally defective and who may not even be on the border line, but who are simply dull, "stupid" children. The dull child is by no means a bad child from an educational point of view. Richard Jefferies, Darwin, Newman, Cecil Rhodes were dull children, who certainly would not have been able, had they been placed under the conditions under which thousands of our children attending the elementary schools have to compete for scholarships, to have carried off one of these blue ribbons.

Yet they were very far from being mentally defective children. Indeed, the more one studies child psychology and the better, through systematic medical inspection and experimental research, we come to understand the school child in all his or her varying types and degrees of mentality, the more convinced we become that it is impossible to differentiate by classes. Differentiation must be a matter of individuality. This the experienced teacher fully realises, but unfortunately the conditions under which he or she works are such that he or she cannot individualise; the system is responsible for the treatment en masse of the children, and so long as it endures in all its antediluvian strictness it is impossible to expect the teacher to discriminate carefully. In the circumstances the task of differentiating is thrown upon the school doctor, who, in the case of the London County Council's medical staff, has an expert psychologist to aid him and a special group of men and women who have excellent experience in mentally defective cases to work with as consultants.

On the other hand are the parents and the children themselves. In present circumstances it is a stigma for a child to attend an M.D. school. In London, for example, the average school-going child refers to these establishments as "soppy schools" and to the children who attend them as "soppies" or softies. It is quite true that the education given in these special schools is of a high type of usefulness: the children are taught matters which are of far greater practical value to them in after-life than the subjects they devote so much time to in the ordinary elementary schools. But the average parent does not see it in this light. He sees in the special school merely a stigma which will cling to the child throughout school life and probably in after-life as well.

The real way out of the difficulty seems to be to make each school wholly self-contained, so that it includes classes for mentally defectives, for physically defectives, for dull children, for myopes, stutterers, deaf and dumb children, and for higher-grade children who are now removed to a central school. There are many arguments in favour of such centralisation; there are some strong arguments against. Whatever may be the ideal, the school doctor has to take existing conditions into account. With these alone can he deal, and while he is waiting for the realisation of the ideal he must so shape his course that he steers clear of the Scylla of the teachers' demands on the one hand and the Charybdis of the parents' prejudices on the other.

In the majority of cases his course is easy enough. He has merely to eliminate the gross physical defects and form an opinion, necessarily qualified, as to the mentality of the children submitted to him for examination. This implies that he has to examine every child presented to him very carefully with regard to physique. Such an examination ought to be stricter and more exhaustive than the usual routine medical inspection. For obviously the children sorted out by the teacher are not average children. They are either dull children, physically defective children with such defects as influence their school life detrimentally, or mentally defective children. Into one or other of these three groups all the children presented to him may be classed, and his duty is obviously to find out in which class or group each child should be placed.

The physical defects that ordinarily account, largely, for dulness of intellect in school are defects of the organs of sense, defects of the respiratory tract, of the nervous system, of the heart, and of nutrition. Adenoids and enlarged tonsils are well known as defects which make a child liable to be classed as dull and stupid. Where they are present a final diagnosis of mental deficiency must be postponed—unless the signs are very clear—until the defect has been treated in a proper manner. Anæmia, fatigue through overstrain, mal-assimilation leading to mal-nutrition either through insufficient food, or, more often, improper feeding, are all defects which can be remedied and which should be remedied before it is possible to obtain a true index of the child's state of mind. Of the defects of the organs of sense the most important and, properly speaking, the only ones that the school doctor will ordinarily meet with, are defects of the eye and of the ear. Partial deafness, unsuspected by the teacher or child, often leads to a diagnosis of "mentally defective child" by the former; astigmatism and myopia may do likewise, although defects of the eye are usually surmised or "spotted" by the teacher. Proper treatment of the ear and the prescription of suitable glasses overcome these difficulties in many cases. Defects of the nervous system are more insidious and not generally suspected by the teacher; where such defects are very patent the child is nearly always unfit for school and should be excluded for treatment. The exception is in the case of "habit neuroses," such as head nodding, twitching of the orbital muscles, and leg and arm movements of an athetotic character. Children subject to such conditions derive little benefit from exclusion; their treatment is educative to a large degree, and the education is best given at the school and not at home.

There remains yet a class of child who, while presenting no definite lesion that can be discovered by inspection, is yet obviously abnormal. In the infant department such children are usually found to be spasmophilics, with a tendency towards fits and convulsions. Such children are often mentally under-developed and ill-nourished in body. In the senior departments a diagnosis of spasmophilia is generally impossible; some other cause for the trouble must be sought for. One of the most common is undoubtedly intestinal parasites. The elaboration of special poisons, engendering fatigue, by worms is now well recognised, while intestinal stasis as a probable cause of fatigue must never be lost sight of. These few hints will show how wide is the field which the school doctor has to investigate before he can definitely proceed to gauge the mentality of the child by one or more of the methods usually employed. A summary of this special examination will be given in the next article.

